# Succession and climatic stochasticity induce long-term decline of a forest browser

**DOI:** 10.1371/journal.pone.0298231

**Published:** 2024-02-27

**Authors:** Eric S. Long, Enoch J. Tham, Ryan P. Ferrer

**Affiliations:** Department of Biology, Seattle Pacific University, Seattle, Washington, United States of America; US Geological Survey, UNITED STATES

## Abstract

Removal of predators and creation of early seral habitat have, in many systems, caused substantial population growth of herbivores. Hyperabundant herbivores, in turn, induce cascading ecosystem effects, but few studies have investigated long-term browser density trends in relation to succession and stochastic climate events. Here, we use annual, empirical population estimates of a forest browser to relate forest succession to long-term decline of an herbivore that prefers early seral habitat. From 2007–2021, concurrent with reduced timber harvest, we used line-transect distance sampling to document annual changes in Columbian black-tailed deer (*Odocoileus hemionus columbianus*) density on a mid-sized (17.3km^2^) predator-free island. We documented successional changes associated with forest aggradation and decreased forage quality for deer: early successional shrub/scrub habitat declined 3.8%/year; timber volume increased 4.5%/year; and canopy coverage increased 2.5%. In 2007–2008, deer densities were the greatest observed (~44/km^2^), but then an historic snowstorm reduced deer density by 39%. From 2010–2021, as forests continued to mature, deer density decreased 4.0%/year, declining to 20 deer/km^2^. Using a multivariate approach to combine habitat variables (i.e., early seral coverage, timber volume, and canopy closure) into a measure of forest maturation, we found a significant negative relationship between deer density and forest aggradation. Thus, consistent with predictions for bottom-up limited browsers, we observed significant annual declines in a deer population throughout an extended period of forest regrowth. Despite declines, deer density on the island exceeds mainland densities, and overbrowsing likely continues to disrupt ecosystem processes.

## Introduction

When control of herbivore populations is reduced, through processes such as predator removal or habitat alteration, browsing and grazing pressure often increase to levels that disrupt ecosystems [1,2]. In forest ecosystems, browsers may benefit from timber management practices that create abundant early seral habitat [3,4], and high browsing pressure in these modified landscapes may alter community composition [5,6]. Stochastic events, such as extreme weather and disease outbreak, may cause drastic, short-term population reductions [7], but especially in the absence of predators, herbivore populations may become hyperabundant and alter ecosystem processes [8].

Throughout much of their range, deer (i.e., species within family Cervidae) have demonstrated population increases, typically relating to predator removal and forest management practices that produce abundant early seral habitat with high forage quality [9]. Selective foraging by hyperabundant deer tends to shift forest plant communities to highly modified, simplified stable states [10–12]. Further, these deer-induced, structural changes in forest ecosystems have cascading effects, influencing above- and below-ground processes [13] and altering habitat for associated animal species [14–17]. For these reasons, deer have been considered ecosystem engineers [18,19] and keystone species [20–22].

Although responses of deer to predators are well-documented [23–25], numeric responses of deer populations to forest succession remains unclear. Habitat usage studies suggest that ungulates use various seral stages disproportionately to their availability. For instance, moose (*Alces alces*), elk (*Cervus canadensis*), and white-tailed deer (*Odocoileus virginianus*) browse extensively on early seral vegetation and may be found abundantly in recent clearcuts or regenerating burns; whereas caribou (*Rangifer tarandus*) are more likely to use mature, old-growth patches that provide adequate cover and lichen for forage [26,27]. Within Poland, Bobek et al. [28] demonstrated an increase in red deer *(Cervus elaphus*) that they attributed to reduced culling, reduced clear-cutting, and increased selective cutting of forests, which increased food and cover availability; however, longitudinal studies demonstrating numeric responses of deer to forest succession remain rare.

Island ecosystems present an ideal, closed environment in which to test the effects of forest succession on deer populations. Much of the research on island deer has focused on the ecological effects of overbrowsing, and because many islands are free of predators, deer populations often greatly exceed mainland densities [29–32]. Although many of these hyperabundant populations of deer are exotic, native populations of island deer also tend to exceed mainland densities. For example, the San Juan and Gulf Island archipelagos in the Salish Sea of Washington (USA) and British Columbia (Canada), respectively, comprise more than 600 islands, and Columbia black-tailed deer (*Odocoileus hemionus columbianus*) are native to this system. Predators, including wolves (*Canis lupus*) and cougars (*Puma concolor*) were historically present on these islands, but these apex predators were extirpated following European settlement in the late 1800s [33]. Concurrently, large-scale timber harvest commenced on the islands, and early seral communities that follow timber harvest have been shown to provide increased forage availability for black-tailed deer in this region [34]. Due to predator removal and substantial timber harvest, black-tailed deer populations are thought to have increased in the region over the past century and typically exceed mainland densities [35–39], although empirical estimates of population density trends are lacking. In recent years, however, timber harvest has declined and forests have matured, which, despite the continued absence of predators, we predict would induce long-term declines in deer population densities. To test the relationship between forest maturation, availability of early seral vegetation, and deer population density, we annually estimated deer population densities from 2007–2021 on an island which last experienced large-scale timber harvest in the mid-1900s. We predicted that canopy closure and forest aggradation would reduce high-quality browse availability, resulting in long-term declines of deer population density.

## Materials and methods

### Study area

**Fig 1:**
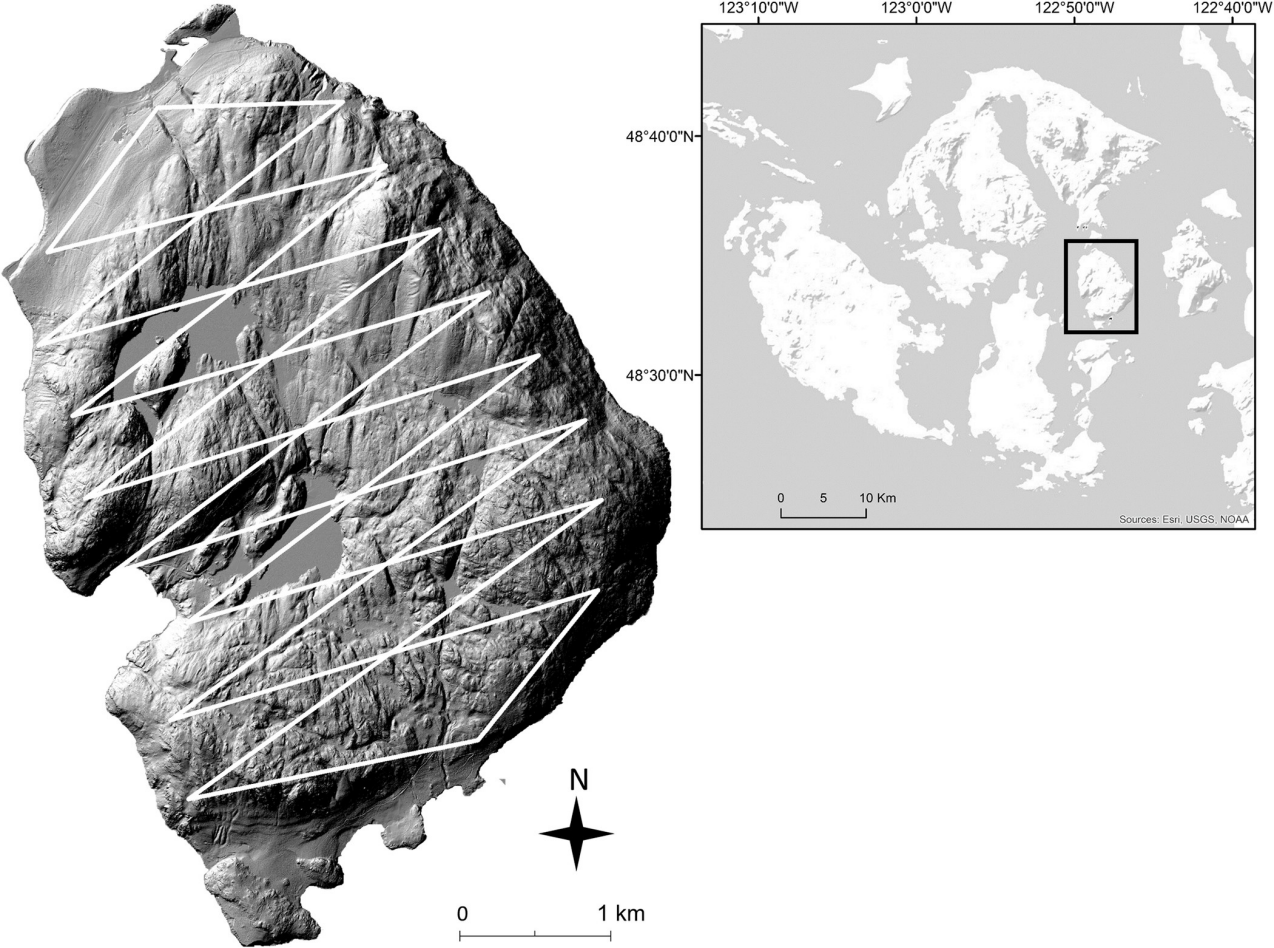
Map of Blakely Island (17.33 km^2^), in the San Juan Archipelago of WA, USA, indicating 16 sawtooth transects used for annual distance sampling surveys of black-tailed deer. Terrain map of San Juan Islands from Esri (Redlands, California, USA); lidar map of Blakely Island accessed from http://pugetsoundlidar.ess.washington.edu.

Blakely Island (17.33 km^2^; 48.56°N, -122.80°W) is a densely forested, mid-sized island in the Salish Sea, Washington, USA (Fig 1). Common tree species include Douglas fir (*Pseudotsuga menziesii*), western hemlock (*Tsuga heterophylla*), and western redcedar (*Thuja plicata*). Associated trees include grand fir (*Abies grandis*), Sitka spruce (*Picea sitchensis*), big-leaf maple (*Acer macrophylum*), and red alder (*Alnus rubra*). Understory consists largely of sword fern (*Polystichum munitum*), bracken fern (*Pteridium aquilinum*), and salal (*Gaultheria shallon*). Additionally, the interior of Blakely Island contains two lakes (27 ha & 30 ha), and numerous smaller palustrine wetlands.

No large predators persist on Blakely Island, and there is limited hunting of deer. No island-specific harvest rate data are available prior to 2013, but from 2013–2021, an average of 16.9 (SE = 1.8) males and 9.9 (SE = 2.1) females were harvested by hunters annually; thus, average annual harvest comprised, on average, less than 7% of the population. Elevation ranges from 0–318 m, and most shorelines are rocky cliffs. Climate is characterized by cool, wet winters and moderate, dry summers. During this study (June 2007 –Dec 2021) average annual precipitation in the region was 75.4 cm; average annual temperature was 10.4°C, with average daily lows of 6.2°C and daily highs of 14.6°C. Snow was rare; with an average of only 4.5 days per year of snow cover >2.5 cm deep. Climate data were accessed from the National Oceanic and Atmospheric Administration’s (NOAA) climate station at Olga, Orcas Island, WA (2.5 km north of Blakely Island) in the San Juan archipelago. When data were unavailable from the station at Olga, we used data from other NOAA stations in the San Juan Islands, including Lopez Island (5.0 km west), Orcas Island (9.7 km northwest), and San Juan Island (15 km west).

Large-scale, commercial timber harvest began on Blakely Island in the 1880s and continued through the mid-1900s. In 1976, a private landowner purchased the interior of Blakely Island from a paper products company, and no commercial forestry operations occurred from 1976–1989. Salvage harvest was conducted from 1990–1994, and commercial patch-cutting occurred from 1995–2005. Approximately 1100 m^3^ of timber/year were harvested from the interior of the island during this 15-year period, and approximately 50,000 saplings were replanted. Prior to our study, and following peak timber harvest in the early 1900s, historical cruise surveys conducted in 1974, 1995, and 2005 suggested substantial net forest aggradation, with annual increases of merchantable timber of 1.5% between 1974–1995 and 1.3% between 1995–2005. During our study, no commercial harvest occurred between 2006–2013, and very small-scale uneven-aged harvest was conducted from 2014–2020, during which only 45 m^3^/year of timber was harvested, and no patches were cleared of canopy. Thus, because no large-scale commercial harvest has been conducted since 2005, we expected net forest aggradation to continue during the period we estimated deer populations, from 2007–2021.

### Forest cover dynamics

We used 3 metrics to quantify forest regeneration and maturation during these recent reduced- and no-harvest periods. First, in 2015, 2016, and 2020, an independent forestry consultant (Rain Shadow Consulting, Eastsound, WA, USA) was commissioned to inventory timber growth. As a part of these monitoring surveys, 24 permanent, randomly located, circular 0.04 ha plots were established. Within each plot, height and diameter at breast height of all trees greater than 10 cm were measured to estimate volume of merchantable timber per ha. Second, we estimated net change of early-successional habitat by accessing Coastal Change Analysis Program (C-CAP) landcover data (30m resolution) from NOAA (www.coast.noaa.gov). Using these remotely sensed raster datasets for Blakely Island from 2001, 2006, 2010, and 2016 (the most recent available year), we identified early successional landcover as pixels coded as grassland and scrub/shrub. We then quantified net changes in early-seral habitat coverage concurrent with reductions in timber harvest. Finally, to quantify canopy closure, we examined aerial photographs of Blakely Island taken in 2006 and 2019, the most recent data available (Pictometry International; www.sanjuanco.com/gis). Using ImageJ software [40], we conducted particle analysis, categorizing all terrestrial pixels on Blakely Island as forested (i.e., covered by canopy) or unforested, to calculate temporal change in total forest cover.

### Deer density dynamics

From 2007–2021, we used line-transect distance sampling to estimate annual population density of black-tailed deer on Blakely Island. Surveys were conducted during daylight hours from July–Aug and consisted of 16 saw-tooth transects (i.e., two sides of a triangle), totaling 44.8 km, and ranging in length from 1.3 to 3.6 km (average = 2.8 km, sd = 0.6 km). A single transect consisted of both the northern and southern edge of each sawtooth (Fig 1). Sawtooth transects [41] were used because they facilitated efficient sampling away from and then back to a central, unpaved access road that longitudinally bisected Blakely Island. Any section of linear transects that intersected a lake was removed, and the total sampling effort (i.e., the total length of transect surveyed) was reduced accordingly.

During surveys, observers used a handheld GPS unit (Garmin GPSMAP 60CSx, Garmin Ltd., Olathe, KS, USA) to walk off-road transect routes, which did not follow any existing trails. Except for 2017, when 2 observers completed a single survey by each surveying half of the transects, each survey was conducted by a single observer, although observers differed from year to year and when multiple surveys were conducted in a single year. When deer were observed, a laser rangefinder (Bushnell Yardage Pro Sport 450, Bushnell Corporation, Overland Park, KS, USA) was used to measure distance in m (*r*) between observer and deer; a handheld compass was used to measure sighting angle (Ѳ) to the deer; and the GPS unit was used to record the observer’s latitude and longitude. From distance and sighting angle measurements, perpendicular distance (d) from the transect line was calculated by

d=r*sin(Ѳ). (1)

Most observations were of single deer, but when groups, typically comprising an adult female and fawn, were observed, group size was recorded.

Density of black-tailed deer on Blakely Island was estimated using program DISTANCE 7.3 (Research Unit for Wildlife Population Assessment, University of St. Andrews). For each year, we compared the fit of 3 models: uniform distribution with cosine adjustment, hazard rate distribution with polynomial adjustment, and half-normal distribution with cosine adjustment. Prior to model fitting, data were truncated by removing the greatest 5% of distance observations. This truncation reduces the number of adjustment terms needed to model the detection function, and observations far from the line contribute little to density estimates [41]. When two separate observers conducted the surveys, we stratified observations by observer to control for potential observer effects [41]. The most parsimonious model for each year, as indicated by Akaike’s Information Criterion (AIC) [42], was then used to estimate annual black-tailed deer density and effective strip width (ESW), which is the estimated distance at which the number of deer missed within that distance equals the number of deer detected beyond that distance.

### Data analysis

From annual deer density estimates, we modeled annual growth rate of the deer population as

$$\ln\left(D\right)=\beta_{0}+\beta_{1}t$$ (2)

where *D* is the density of deer, *t* is the year of the estimate, and an annual (i.e., exponential) growth rate of the deer population (*r*_*d*_) was estimated as

$$r_{d}=-(1-e^{\beta_{1}}).$$ (3)

We then used linear regression modelling to test whether annual deer population estimates demonstrated significant declines concurrent with forest maturation.

To relate deer density directly to forest aggradation, we estimated annual growth rates for each of the 3 habitat variables (timber volume, shrub/scrub habitat coverage, and percent canopy coverage) by

$$r_{h}={\left(\frac{h_{t}}{h_{0}}\right)}^{t_{h}}-1,$$ (4)

where *r*_*h*_ is the annual rate of growth for each habitat variable (*h*), *h*_*0*_ is the first available estimate for each habitat variable during our study, *h*_*t*_ is the last available estimate for each habitat *variable*, and *t*_*h*_ is the total number of years for which each habitat variable was available. We used these annual estimates of growth for each habitat variable to estimate habitat values over the period for which we estimated deer population density (2007–2021). Because the habitat variables were highly correlated, we conducted a principal components analysis of the natural log of each of the 3 habitat variables to model forest aggradation, using the FactoMineR package [43] in Program R (v4.3.2, R Core Team 2023). The first principal component (PC1, a linear combination of variables that related positively to timber volume increase, early seral coverage decrease, and canopy coverage increase) was then used as the independent variable in a linear regression relating deer density to forest aggradation.

## Results

### Forest cover dynamics

Timber monitoring surveys conducted from 2015–2020 indicated net forest aggradation. From 2015–2016, timber volume increased from 3180–3420 m^3^/km^2^, and by 2020 volume increased to 3990 m^3^/km^2^. This growth represents a 26% increase in forest volume during the 5-year monitoring period, corresponding to an annual volumetric growth rate of 4.5%.

**Fig 2:**
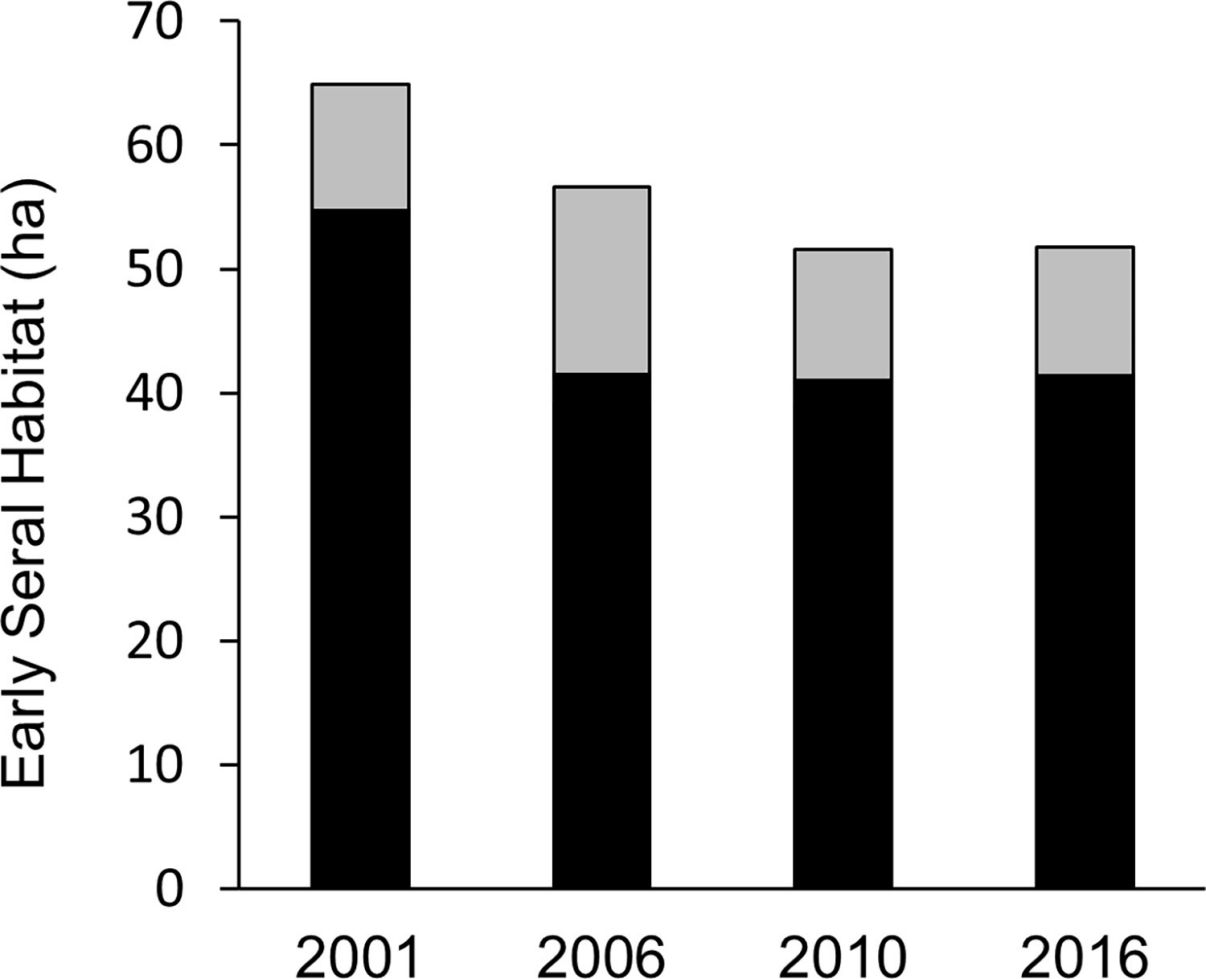
Loss of early seral habitat (grassland = black, scrub/shrub = grey) on Blakely Island, Washington, USA, as calculated from remotely sensed, 30m resolution landcover maps (Coastal Change Analysis Program, NOAA).

Consistent with the forest maturation indicated by monitoring surveys, NOAA C-CAP landcover data from 2001–2016 indicated an overall 20.2% loss (i.e., 1.5%/year) of early successional habitat (Fig 2). From 2001–2006, this pattern corresponded primarily to a 24.2% decrease in grassland. However, from 2006–2016, concurrent with the beginning of our study, grassland coverage remained consistent, but scrub/shrub habitat decreased by 31.5% (i.e., 3.7%/year).

Similarly, comparison of aerial photographs of Blakely Island taken in 2006 and 2019 also suggests a pattern of net canopy closure, as forest coverage increased from 90.74% to 93.21%, corresponding to a net gain of 0.40 km^2^ of forest coverage. Overall, these data indicate that, during our study, net forest aggradation and maturation occurred, such that timber volume increased, early successional habitat decreased, and forest canopy closed.

### Deer density dynamics

Due to logistical constraints, we were unable to conduct black-tailed deer surveys in 2012 and 2020; otherwise in a given year, all 16 distance-sampling transects were surveyed either once (n = 5) or twice (n = 8; Table 1). Although we tested multiple models of detection function (i.e., half-normal, hazard rate, and uniform) in each year, and used lowest AIC to select the most parsimonious model within a year, density estimates were not particularly sensitive to model choice. Average deviation of estimated deer density from the second ranked model compared to the highest ranked model was 5.1% (sd = 3.1%), and average deviation of all tested models from chosen model was 8.2% (sd = 7.1%;). Thus, regardless of the specific model chosen, density estimates within a year were relatively consistent.

**Table 1:** Distance sampling results for annual, off-trail, transect-based surveys to estimate population density of black-tailed deer on Blakely Island, Washington, USA.

Year	N Surveys	Deer obs.	Density (km^-2^)	Density 95% CI	CV	ESW (m)	Model
2007	2	148	38.6	27.5–54.2	0.17	19.4	Hazard rate (polynomial)
2008	2	172	49.6	39.3–62.5	0.12	18.5	Uniform (cosine)
2009	2	111	26.9	20.9–34.6	0.13	21.3	Half-normal (cosine)
2010	1	44	31.0	21.3–45.2	0.19	15.1	Uniform (cosine)
2011	1	74	30.6	19.4–48.1	0.22	25.2	Half-normal (cosine)
2013	2	60	23.3	12.1–44.9	0.34	14.4	Hazard rate (polynomial)
2014	1	40	31.7	18.4–54.6	0.27	15.0	Uniform (cosine)
2015	2	88	24.9	17.4–34.5	0.18	18.9	Uniform (cosine)
2016	2	84	25.3	17.7–36.1	0.18	20.3	Hazard rate (polynomial)
2017	1	36	24.7	16.6–36.7	0.19	19.5	Uniform (cosine)
2018	1	42	16.9	9.9–28.7	0.26	27.1	Uniform (cosine)
2019	2	69	25.2	17.4–36.4	0.19	18.9	Uniform (cosine)
2021	2	62	19.9	14.9–26.5	0.15	20.7	Hazard rate (polynomial)

Surveys were conducted once (n = 5) or twice (n = 8) per summer from 2007–2021. ESW is the effective strip width. Model is the most parsimonious model tested, as determined by AIC, with model adjustment terms in parentheses.

Initial distance sampling estimates of deer density on Blakely Island, conducted in summer 2007 and 2008, indicated the highest population densities recorded during the study (Table 1). However, in Dec 2008, an abnormally intense winter storm resulted in 29.3 cm of snowfall, maximum snow depth of 25.4 cm, and 15 days of snow cover deeper than 2.5 cm. For comparison, the second most intense snowstorm during the study occurred in Jan 2020, resulting in 27.8 cm of snowfall and maximum depth of 20.3 cm, but snow cover only persisted for 4 days. Climate records for the region extend to 1891, and only twice do records indicate snow persisting for more than 15 days (17 days in 1993 and 22 days in 1950), although maximum snow depth during those storms was only 10.2 cm and 12.7 cm, respectively. Thus, relative to the Dec 2008 storm, no storm recorded in the past 130 years has resulted in deeper snow that persisted longer.

Following the Dec 2008 snowstorm, density estimates in 2009 suggested a drastic, 39% decline in the deer population from pre-storm average densities. In 2010, point estimates suggested that the deer population recovered slightly, but this density remained 30% lower than average pre-storm density (Table 1).

Since 2010, deer populations have not recovered to pre-storm densities; rather, they have demonstrated a significant decline of 4.0%/year, such that

ln(D)=85.38(±28.03)−0.041(±0.014)t, (5)

where *D* is deer density per km^2^, *t* is year of population estimate, and values in parentheses are SE of coefficients (R^2^ = 0.52, p = 0.019, n = 10). The most recent density estimates in 2021 suggest a 36% decline from 2010 density and a 55% decline from initial, average pre-storm density.

In multidimensional analysis of habitat variables, PC1 modeled forest aggradation (i.e., increase in timber volume and canopy closure, decrease in shrub/scrub coverage). Linear regression indicated a significant negative relationship between PC1 and deer density (D), such that

**Fig 3:**
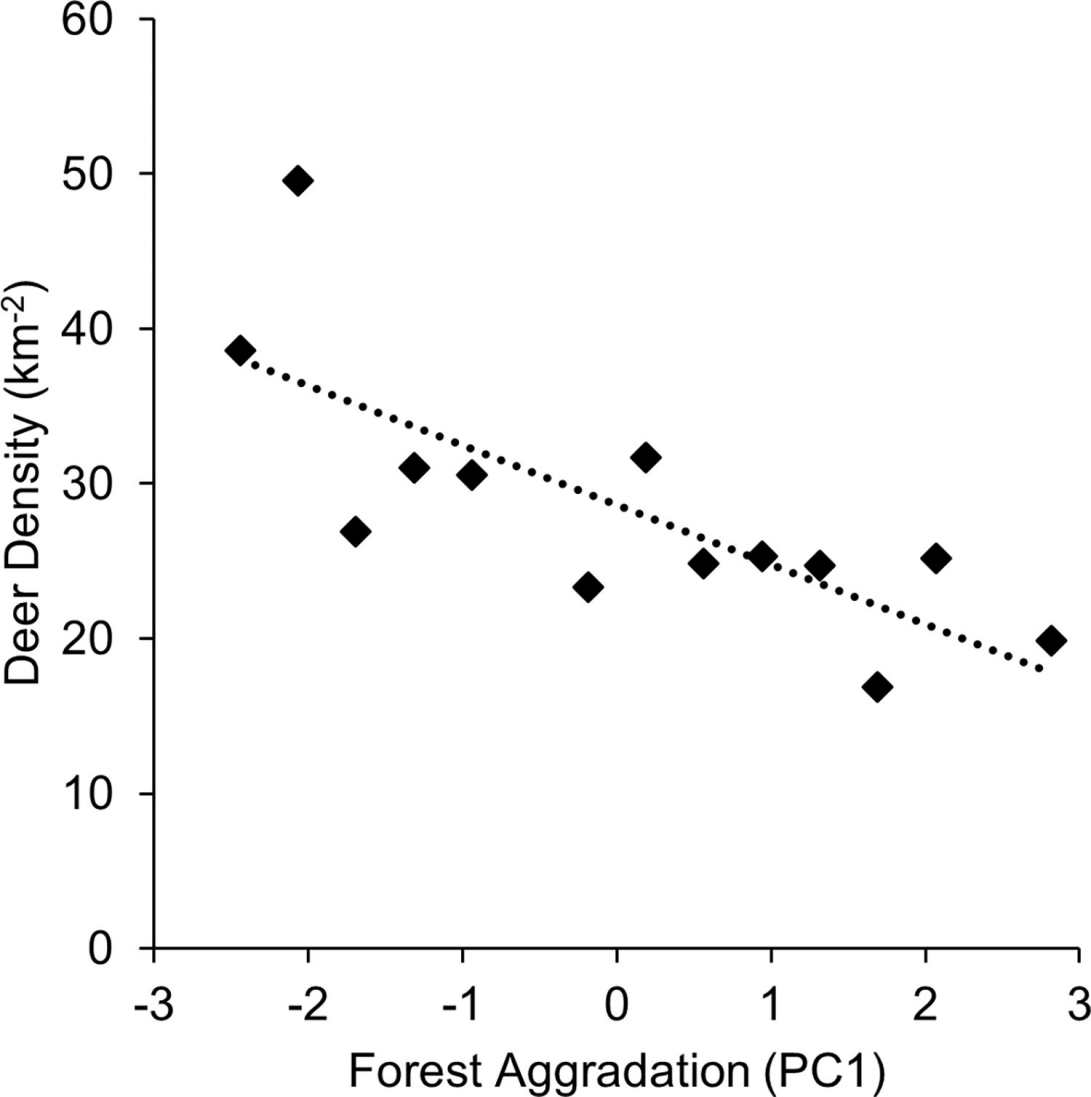
Relationship between forest aggradation and black-tailed deer density on Blakely Island, WA. PC1 is a linear combination of 3 habitat variables and indicates a decline in early seral coverage, increase in timber volume, and closure of forest canopy.

D=28.63(±1.57)−3.86(±0.98)*PC1, (6)

suggesting that deer density declined as trees matured, canopy closed, and early seral habitat declined (R^2^ = 0.59, p = 0.002, n = 13; Fig 3). When analysis was limited to years following the snowstorm (2010–2021), we found a similar significant negative relationship between forest aggradation and deer (R^2^ = 0.55, p = 0.014, n = 10).

## Discussion

Following cessation of large-scale timber harvest on Blakley Island in the mid-1900s, the forest has demonstrated net aggradation. Patch-cut timber harvesting resumed from 1990–2005, and these patches likely improved forage quality for black-tailed deer on the island; however, since 2005, very little timber harvest has occurred, and our data suggest that forest succession has resulted in loss of early seral habitats and gain of closed canopy stands. In northern California, Bose et al. [44] showed that black-tailed deer select open-canopy forest patches. Further, working on the mainland of western Washington, Ulappa et al. [34] demonstrated that forage suitable for black-tailed deer was approximately 25 times greater in early successional patches relative to closed-canopy forest. Thus, especially in the absence of predators, succession-induced decline in forage availability seems to intensify bottom-up control, which is predicted to result in long-term decline of black-tailed deer in the region [45]. Consistent with this prediction, we demonstrated a negative relationship between deer densities and forest aggradation and a significant annual decline in deer populations as the forest ecosystem matured and early seral patches converted to closed canopy forest. Working on Northwest Bay, Vancouver Island, Smith [46], in an unpublished thesis, used hunter success rates and direct, uncorrected deer counts to suggest declines in black-tailed deer density related to hunting, winter severity, and seral succession following fire and logging; however, his methodology precluded empirical estimation of deer population density.

Although deer density on Blakely Island decreased as timber-cutting slowed and forests matured, this pattern is likely not ubiquitous across the range of black-tailed deer, especially in regions with regular, severe winters [27]. Deep snow covers available forage in early seral habitats; thus, in snowbelt regions black-tailed deer tend to select mature forest habitats relative to recent clearcuts, especially in winter [47–49]. Based on these habitat usage patterns, in regions with regular deep snow, canopy-clearing silviculture may decrease population size of deer.

Deep and persistent snow cover experienced by high-density populations of deer, especially those not accustomed to severe winters, may result in substantial mortality. In addition to covering available forage [50,51], deep snow increases energetic costs of locomotion and thermoregulation [52], challenges that may be exacerbated by dwarfed body size, which is evidenced by some island populations of deer, such as the deer on Blakely Island [39,53]. Further, herbivores in high-density, predator-free populations have been shown to experience disproportionately negative effects from stochastic events, a pattern which likely relates to low resource availability and poor body condition [7,54,55]. Even in areas without regular deep snow, as in our study system, deer tend to reduce winter movements, which is likely a strategy to reduce energy expenditure during a period of poor forage quality [56]. In systems with predators, increased snow depth may increase vulnerability of deer to predation [57,58], but by limiting forage availability, unusually severe winters may also cause mass mortality of bottom-up regulated populations of deer. For instance, while studying a hyperabundant population of sika deer (*Cervus nippon*) on a predator-free island in Japan, Takatsuki et al. [59] observed a starvation-induced die-off of 42.6%, following an extremely severe winter. Although climatic warming will reduce snowfall in many regions, the effects of climate change on frequency and intensity of winter storms accompanied by deep snow and ice remains unclear, as some regions are expected to experience increases in winter storms and other areas expected to experience decreases [60]. As shown here, severe storms may drastically reduce herbivore populations, especially where populations are not locally adapted to deep snow; further, populations may not quickly recover from such stochastic disturbances.

Although the population of deer on Blakely Island is decreasing, deer density on the island still exceeds black-tailed deer density on the mainland and on large islands with predators. At the beginning of our study, prior to the Dec 2008 snow-induced mortality event, population density averaged 44 deer/km^2^, but the most recent densityf estimates from 2021 suggest density was reduced to approximately 20 deer/km^2^. Using mark-resight surveys in SW Oregon (USA), Smith [61] estimated approximately 13 black-tailed deer/km^2^, and in suburban NW Oregon (USA), Happe [62], using spotlight counts, estimated approximately 9 deer/km^2^. Bender et al. [63] estimated 2.7 black-tailed deer/km^2^ in rural SW Washington (USA), although density estimation techniques were not described for that study. Within the Nimpkish Valley of northern Vancouver Island (British Columbia, Canada; 31,284 km^2^, 375 km NW of Blakely Island), Hatter and Janz [36] used spotlight counts to report a range of black-tailed deer densities from 2.8–14.8/km^2^, attributing the wide range of density estimates to temporal variation in severity of wolf predation during a period of wolf control. Wingard [64] used road-based distance sampling to estimate 6 deer/km^2^ on Whidbey Island (Washington, USA; 437 km^2^, 18 km SE of Blakely Island), an island on which coyotes (*Canis latrans*) are present.

Black-tailed deer density on predator-free islands tends to be greater than mainland density, and high browsing pressure of hyperabundant island deer populations in the region has led to cascading ecological effects [38,65–67]. Although empirical estimates are lacking, Martin and Baltzinger [68] estimated a mean black-tailed deer density of 20 deer/km^2^ for the Haida Gwaii Archipelago (British Columbia, Canada), and they note severe lack of western redcedar regeneration throughout much of these Islands. On East Limestone Island (0.48 km^2^) in the Haida Gwaii Islands, Daufresne and Martin [69] censused 16 deer, equating to 33 deer/km.^2^ Similarly on Piers Island (1.01km^2^) in the Gulf Island Archipelago, Martin et al. [38] censused 22 deer, equating to 22 deer/km^2^. Then, by calculating fecal pellet density on Piers Island, Martin et al. [38] used the known ratio of fecal density to deer density on Piers Island to convert fecal density to deer density on 7 additional islands in the Gulf and San Juan Archipelagos, with estimates ranging from 13–114 deer/km^2^ (median = 27.5). Martin et al. [38] concluded that even at densities of ~10 deer/km^2^, vegetation was overbrowsed, and bird diversity was depleted. Similarly, Martin and Baltzinger [68] noted that deer density as low as 4 deer/km^2^ may be required for regeneration of western redcedar in the Haida Gwaii Islands.

In other forested ecosystems, high browsing pressure from deer has been shown to slow or alter secondary succession patterns. For instance, DiTommaso et al. [70] demonstrated that white-tailed deer reduced recruitment of woody species, and selective browsing favored recruitment of short-lived and invasive species. Van Deelen et al. [71] suggested that forest succession may continue despite high white-tailed deer densities, but deer herbivory will favor the establishment of unpalatable species. We suspect that similar patterns are occurring with black-tailed deer herbivory within the San Juan Archipelago. For instance, Arcese et al. [72] demonstrated that palatable plant coverage was 92% less on high deer-density islands within the Salish Sea. On Blakely Island, anecdotal evidence suggests that palatable tree species, such as Douglas firs and western redcedars, experience high browsing pressure and have low recruitment; whereas less palatable species, such as western hemlock, have relatively high regeneration rates. Further research is needed to quantify the interactive effects of deer herbivory and tree recruitment, but if deer continue to shift communities toward unpalatable species, we anticipate further declines in deer density as forest communities become increasingly dominated by relatively unpalatable species. Further, if deer herbivory induces a transition to western hemlock-dominated forest, then browsing pressure may interact with climate change to increase the likelihood of a stand-replacing fire. Regional climate change predictions suggest that summer drought conditions will intensify, increasing the probability of fire [73], and western hemlock is more fire prone than Douglas fir and western redcedar [74].

During the 14 years of this study, hyperabundant, bottom-up regulated black-tailed deer on Blakely Island demonstrated long-term population decline of over 50% as timber harvest slowed, forests matured, early successional habitat was lost, and canopies closed. An extreme snowstorm caused severe reduction in the population, and deer density never returned to pre-storm density, likely due to inadequate food resources. Despite forest succession and climatic stochasticity, deer density on this predator-free island remains greater than on the mainland and, like other islands in the region, likely continues to exceed density at which island ecosystems may recover from a history of overbrowsing.
